# A phase II trial of erlotinib monotherapy for pretreated elderly patients with advanced *EGFR* wild-type non-small cell lung cancer

**DOI:** 10.1186/s13104-015-1214-9

**Published:** 2015-06-05

**Authors:** Hiroyuki Minemura, Hiroshi Yokouchi, Keisuke Azuma, Ken-ichiro Hirai, Satoko Sekine, Kengo Oshima, Kenya Kanazawa, Yoshinori Tanino, Yayoi Inokoshi, Taeko Ishii, Yutaka Katsuura, Akio Oishi, Takashi Ishida, Mitsuru Munakata

**Affiliations:** Department of Pulmonary Medicine, Fukushima Medical University, 1 Hikariga-oka, Fukushima, 960-1295 Japan; Department of Pulmonary Medicine, Saiseikai Fukushima General Hospital, 25 Omori Aza Shimo-harada, Fukushima, 960-1101 Japan; Department of Thoracic Surgery, Fukushima Red Cross Hospital, 11-31 Irie-cho, Fukushima, 960-8530 Japan; Clinical Oncology Center, Fukushima Medical University Hospital, 1 Hikariga-oka, Fukushima, 960-1295 Japan

**Keywords:** Non-small cell lung cancer, Erlotinib, Elderly, *EGFR* wild-type, PCR-invader

## Abstract

**Background:**

Erlotinib is an epidermal growth factor receptor (EGFR) tyrosine kinase inhibitor, which is an effective treatment for patients with non-small cell lung cancer (NSCLC), especially those harboring activating *EGFR* mutations. A previous phase III trial suggested that patients with *EGFR* wild-type (*EGFR*-wt) NSCLC or elderly patients with disease progression after cytotoxic chemotherapy might benefit from erlotinib monotherapy. However, few studies have prospectively evaluated the efficacy and safety of second- or third-line erlotinib monotherapy for elderly patients with *EGFR*-wt advanced or recurrent NSCLC.

**Methods:**

Pretreated patients aged ≥70 years with *EGFR*-wt stage IIIB/IV NSCLC or those with postoperative recurrence were enrolled and received oral erlotinib at a dose of 150 mg/day until disease progression. Primary outcome was the objective response rate (ORR). Secondary end points included the disease control rate (DCR), progression-free survival (PFS), overall survival (OS), and toxicity profile.

**Results:**

This study was terminated early because of the results from a Japanese phase III trial (DELTA trial). Sixteen patients were enrolled between April 2010 and May 2013. The median age was 78 years (range 70–84 years). Six patients were female. Five patients had an Eastern Cooperative Oncology Group performance status of 0. Eleven (69%) patients had adenocarcinoma. Fifteen (94%) patients were treated with erlotinib as a second-line therapy. The ORR was 0% [95% confidence interval (CI) 0–17.1]. DCR was 56.3% (95% CI 33.2–76.9). The median PFS and OS were 1.7 months (95% CI 1.3–2.2) and 7.2 months (95% CI 5.6–8.7), respectively. The most commonly occurring adverse events included acneiform eruption (31.3%) and skin rash (25.0%). One patient developed grade 3 interstitial lung disease, which improved following steroid therapy.

**Conclusions:**

In pretreated elderly patients with advanced or recurrent *EGFR*-wt NSCLC, daily oral erlotinib was well tolerated; however, administration of the drug should not be considered as a second line therapy.

**Trial registration:** University Hospital Medical Information Network (UMIN) Clinical Trials Registry UMIN000004561 (Date of registration: November 15th, 2010)

**Electronic supplementary material:**

The online version of this article (doi:10.1186/s13104-015-1214-9) contains supplementary material, which is available to authorized users.

## Background

In industrialized countries, increasing longevity and declining fertility rates are shifting the age distribution of populations toward older age groups. Thus, the prevalence and incidence of various diseases are increasing, and approximately 50% of patients at diagnosis of non-small cell lung cancer (NSCLC) are >70 years old [1]. Studies on various treatments including vinorelbine [2], gemcitabine [3], and docetaxel [4] as first-line therapy for this population have been conducted. However, the clinical outcomes in terms of tumor response and survival were not satisfactory because of the limited efficacy of these monotherapies. Prospective studies of second-line treatments for this patient population are limited. Thus, exploration of an optimal treatment strategy for elderly patients with NSCLC, as either first-line or second-line therapy is required.

Epidermal growth factor receptor (EGFR) tyrosine kinase inhibitor (TKI) treatment, which is less toxic than cytotoxic chemotherapy, is the standard treatment option for pretreated patients with advanced NSCLC [5, 6]. In addition, first-line gefitinib, an EGFR-TKI, is an effective and feasible treatment for elderly advanced NSCLC patients with activating mutations who were relatively ineligible for standard chemotherapy [7].

BR.21 was a randomized phase III trial comparing EGFR-TKI erlotinib with the best supportive care for pretreated patients with advanced NSCLC. The post hoc subgroup analysis of the trial showed that elderly and EGFR status unknown patients who underwent treatment with erlotinib acquired substantial survival benefit and improved quality of life [8]. Further subgroup analysis showed that the patients with *EGFR* wild-type (*EGFR*-wt) may also benefit from erlotinib [9]. However, prospective investigation of the clinical benefit of erlotinib for pretreated elderly patients with *EGFR*-wt advanced or recurrent NSCLC has not been reported.

We conducted a prospective phase II trial to evaluate the efficacy and tolerability of erlotinib in pretreated elderly patients with *EGFR*-wt advanced or recurrent NSCLC.

## Methods

### Patients and methods

Eligibility criteria included: age ≥70 years; pathologically or cytologically proven NSCLC; measurable tumor sites according to the Response Evaluation Criteria in Solid Tumors (RECIST) guideline version 1.1; an Eastern Cooperative Oncology Group performance status of 0–2; no activating *EGFR* gene mutations (exon 18, 19, 20 and 21); history of 1–2 regimens of systemic chemotherapy; stage IIIB or IV NSCLC, or postoperative recurrence; EGFR-TKI treatment naïve; and appropriate organ function. Required laboratory criteria were white blood cell count >3,000/mm^3^, neutrophil count >1,500/mm^3^, platelet count >100,000/mm^3^, hemoglobin >9.0 g/dL, aspartate aminotransferase (AST) or alanine aminotransferase (ALT) <1.5-fold the upper limit of normal (ULN), total bilirubin <1.5 mg/dL, and serum creatinine <1.5-fold the ULN.

Patients who had received chemotherapy within 4 weeks of trial registration, those who had undergone chest radiotherapy within 12 weeks of trial registration, and those with interstitial lung disease (ILD) were excluded.

Baseline pretreatment evaluations included a physical examination, chest and abdominal computed tomography (CT), brain magnetic resonance imaging (MRI), and radionuclide bone scintigraphy or positron emission tomography. All images were taken within 4 weeks of trial registration.

All enrolled patients provided written informed consent. This study was performed in accordance with the Helsinki Declaration of the World Medical Association, and the protocol was approved by the Institutional Review Board of each participating institution. The main Institutional Review Board that approved our trial was that of Fukushima Medical University, with an approval number of 917 on February 26th, 2009. This study was subsequently registered with the University Hospital Medical Information Network (UMIN) Clinical Trials Registry; identification number, UMIN 000004561.

### Assessment of tumor *EGFR* gene mutation status

*EGFR* gene mutation analysis was performed using invasive signal amplification reaction using a structure-specific 5′ nuclease with a polymerase chain reaction (PCR) product (PCR-invader) [10].

### Assessment of antitumor activity, survival measures, and toxicity

Response Evaluation Criteria in Solid Tumors **(**RECIST) version 1.1 was used to evaluate tumor response. CT to assess target or non-target lesions was conducted every 4 weeks (MRI for brain, where appropriate, was also conducted). A complete response (CR) was defined as the disappearance of all target and non-target lesions. A partial response (PR) was defined as at least a 30% decrease in the sum of the diameters of the target lesions compared with the baseline sum of the longest diameters, with no progression of non-target lesions and no new lesions [11]. Stable disease (SD) was defined as no disease progression or tumor growth for at least 6 weeks. Progressive disease (PD) was defined as a 20% increase of the sum of measurable lesions, unequivocal progression of non-measurable lesions, or the appearance of new disease despite treatment. Objective response rate (ORR) was defined as the proportion of patients whose best response was either CR or PR in the intent-to-treat (ITT) analysis. Disease control rate (DCR) was defined as the proportion of the patients whose best response was CR, PR or SD in the ITT analysis. Progression-free survival (PFS) was defined as the time from registration to objective tumor progression or death from any cause, and overall survival (OS) was the time from registration until death. Responses were confirmed by the central review board. All toxicities were graded according to the National Cancer Institute Common Terminology Criteria for Adverse Events (version 3.0).

### Treatment regimen

Erlotinib was administrated orally at a dose of 150 mg/day, and was discontinued if patients developed ≥grade 2 toxicities. For skin disorders, patients who recovered from grade 2 toxicities could restart erlotinib on the same dose, whereas in those who improved from grade 3 to grade 1 skin disorders, the dose was reduced to 100 mg/day. Erlotinib treatment was discontinued in cases where the following conditions occurred: (1) disease progression; (2) withdrawal of informed consent; (3) development of grade 4 non-hematologic toxicity; and (4) any ILD grade. Local therapies such as thoracic surgery/radiotherapy and other systemic anti-cancer treatments were not permitted during the trial.

### Statistical analyses

To determine the number of patients required to power the study, we assumed the lower limit of ORR to be 10.0% based on the ORR of erlotinib for elderly patients with unknown or wild-type status of *EGFR* mutation, which was reported previously as 7.0–10.0% [8, 9, 12, 13], and that an ORR of 25.0% in eligible patients would indicate potential usefulness. With an alpha value of 0.05 and 80% power, we estimated that a total of 40 patients would be needed.

The PFS and OS were estimated using the Kaplan–Meier method. The 95% confidence interval (CI) of the response rates were evaluated using the Clopper–Pearson method. The statistical analyses were performed using SPSS software, version 20 (IBM Corporation, Armonk, NY, USA). A *p* value of <0.05 was considered statistically significant.

## Results

### Patient characteristics

**Figure 1:**
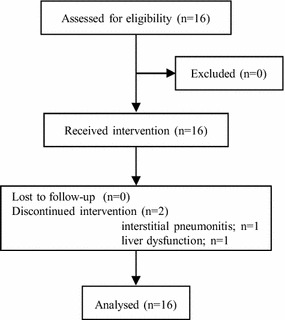
CONSORT diagram.

This trial was terminated when the results of the DELTA trial were presented at the American Society of Clinical Oncology (ASCO) Annual Meeting in 2013 [14]. Between April 2010 and May 2013, 16 patients were enrolled. CONSORT diagram is shown in Figure 1 (Additional file 1). All patients assessed for eligibility were assigned to receive erlotinib and analyzed. The patients’ baseline characteristics are listed in Table 1. The median age was 78 years (range 70–84), six patients were female, and five patients had a performance status of 0. Eleven patients had adenocarcinoma, two patients had squamous cell carcinoma and one patient had adenosquamous carcinoma. Fifteen (94%) patients were treated with erlotinib as a second-line therapy.

**Table 1:** Patient characteristics

	Patients	
	n	%
Age, years [median (range)]	78 (70–84)	
Sex		
Female	6	37.5
Male	10	62.5
ECOG PS		
0	5	31.3
1	5	31.3
2	6	37.4
Histology		
Adenocarcinoma	11	68.8
Squamous cell carcinoma	2	12.5
Adenosquamous cell carcinoma	1	6.2
NOS	2	12.5
Clinical stage (TNM ver.7)		
IIIB	4	25.0
IV	11	68.8
Post operative recurrence	1	6.2
No. of prior chemotherapy regimen		
1	15	93.8
2	1	6.2

*ECOG PS* Eastern Cooperative Oncology Group performance status, *TNM* tumor-node-metastasis, *NOS* not otherwise specified.

### Efficacy

**Figure 2:**
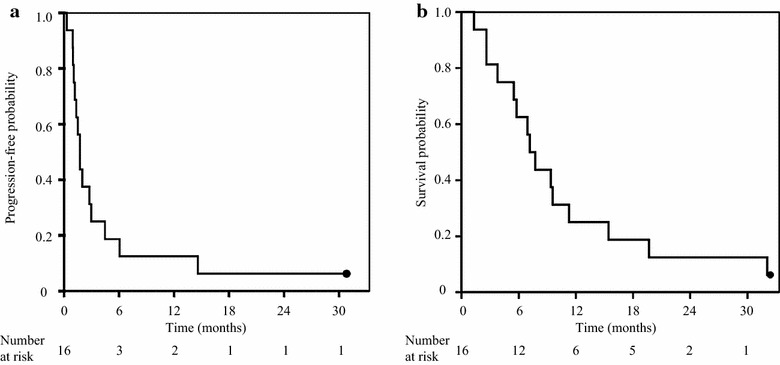
Survival outcomes after erlotinib treatment. Kaplan–Meier estimates of progression-free survival (**a**) and overall survival (**b**). *Dot* censored case at the data cut-off point.

All 16 patients were evaluable for response. The ORR and DCR were 0.0% (95% CI 0.0–17.1%) and 56.3% (95% CI 33.2–76.9%), respectively (Table 2). With a median follow-up time of 7.4 months (range 1.3–32.0 months), 15 (94%) patients experienced disease progression or died. Median PFS and OS were 1.7 months (95% CI 1.3–2.2 months) and 7.2 months (95% CI 5.6–8.7 months), respectively (Figure 2).

**Table 2:** Objective response (RECIST version 1.1)

	n	%
Number of patients evaluated	16	
CR	0	0
PR	0	0
SD	9	56.3
PD	7	43.7

*RECIST* response evaluation criteria in solid tumors, *CR* complete response, *PR* partial response, *SD* stable disease, *PD* progressive disease.

### Safety

The incidence of treatment-related adverse events is summarized in Table 3. The most common adverse event was grade 1/2 acneiform eruption (31.3%, n = 5 patients), followed by skin rash (25.0%, n = 4 patients). All patients with adverse skin reactions continued erlotinib, except for those with grade 2 toxicity that required temporary discontinuation according to the protocol. Grade 3/4 non-hematologic toxicities occurred in two patients, one with elevated AST and ALT levels (6.3%), and one who developed ILD (6.3%). The patient with ILD received systemic steroid therapy and showed full recovery, although erlotinib treatment was discontinued. The patient with elevated AST/ALT levels, recovered on erlotinib discontinuation, and did not recommence treatment.

**Table 3:** Adverse events (CTCAE version 3.0)

Adverse event	Grade ½, n (%)	Grade 3, n (%)	Grade 4, n (%)
Anemia	1 (6.3)	0 (0)	0 (0)
Elevation of AST	2 (12.5)	1 (6.3)	0 (0)
Elevation of ALT	2 (12.5)	1 (6.3)	0 (0)
Elevation of creatinine	1 (6.3)	0 (0)	0 (0)
Albuminuria	1 (6.3)	0 (0)	0 (0)
Interstitial lung disease	0 (0)	1 (6.3)	0 (0)
Acneiform eruption	5 (31.3)	0 (0)	0 (0)
Rash	4 (25.0)	0 (0)	0 (0)
Dry skin	1 (6.3)	0 (0)	0 (0)
Paronychia	1 (6.3)	0 (0)	0 (0)
Stomatitis	3 (18.8)	0 (0)	0 (0)
Glossitis	1 (6.3)	0 (0)	0 (0)
Diarrhea	2 (12.5)	0 (0)	0 (0)
Anorexia	2 (12.5)	0 (0)	0 (0)
Malaise	1 (6.3)	0 (0)	0 (0)
Hypotension	1 (6.3)	0 (0)	0 (0)
Dysgeusia	1 (6.3)	0 (0)	0 (0)

*CTCAE* Common Terminology Criteria for Adverse Event, *AST* aspartate aminotransferase, *ALT* alanine aminotransferase.

## Discussion

To the best of our knowledge, this is the first prospective trial to evaluate erlotinib monotherapy for pretreated elderly patients with *EGFR*-wt NSCLC, although the study was terminated early. In this trial, the ORR was lower than expected.

Previous clinical trials reported the effectiveness of erlotinib for patients with *EGFR*-wt NSCLC. Subgroup analysis of BR.21 demonstrated that 7% of non-squamous *EGFR*-wt NSCLC patients responded to erlotinib and 8% of elderly, EGFR status- unknown NSCLC patients responded to erlotinib [8, 9]. The SATURN trial investigated the efficacy of erlotinib as a switch maintenance therapy following four cycles of platinum-based chemotherapy. Compared with the placebo in the trial, erlotinib prolonged the survival of patients with *EGFR*-wt NSCLC [12.4 vs 8.7 months, HR 0.65 (95% CI 0.48–0.87); *p* = 0.0041] [15, 16]. In a sharp contrast, several other prospective studies [17, 18] demonstrated that the ORR of erlotinib monotherapy in a second-line setting was <5%. In the present study, we believe there are several reasons for the low ORR. First, the study evaluated elderly patients, who most likely have potentially poorer organ function and a lower performance status compared with younger patients. Second, EGFR status was determined in this study by PCR-invader assay, whereas direct sequencing was used in the BR.21 trial. The sensitivity and specificity of direct sequencing has recently been confirmed as being lower than those of PCR-based methods [19–21]. The high specificity of the PCR-invader assay may be associated with a more accurate assessment of negative *EGFR* mutation status, resulting in a lower ORR in the current study.

The slow accrual of this study was because of the release of data from the TAILOR trial [22]. In addition, the study was finally terminated following the disclosure of data from the DELTA trial [14], which recruited Japanese NSCLC patients following the TAILOR trial. In both studies, erlotinib failed to show any improvement in PFS compared with docetaxel in pretreated advanced *EGFR*-wt NSCLC patients aged ≥20 years. A recent meta-analysis also demonstrated that in patients with advanced *EGFR*-wt NSCLC, conventional chemotherapy was associated with better improvement of PFS compared with first-generation EGFR-TKIs such as gefitinib and erlotinib [23]. Therefore, we believe early termination of this study was appropriate.

The frequency of skin-related adverse events in the current study was comparable to the frequency of skin disorders reported in a large-scale erlotinib-treated Japanese cohort [24]. In the current study, all adverse events improved with short-term discontinuation, and all but two patients with grade 3/4 events recommenced erlotinib treatment. The large-scale Japanese study reported that the incidence of ILD was 4.5%, and ILD-related death occurred in 1.6% of patients. The higher incidence of ILD in the current study was most likely because of the small patient population. However, there were no cases of ILD-related death.

## Conclusions

Erlotinib monotherapy was well tolerated in pretreated elderly patients with *EGFR*-wt advanced or recurrent NSCLC. However, erlotinib should not be considered as a second line therapy for pretreated *EGFR*-wt elderly NSCLC patients.

## Additional file

Additional file 1. CONSORT 2010 checklist of information to include when reporting a randomised trial.
